# Complete mitochondrial genome of near threatened butter Catfish *Ompok bimaculatus* (Siluriformes: Siluridae)

**DOI:** 10.1080/23802359.2017.1334520

**Published:** 2017-05-30

**Authors:** Anindya Sundar Barman, Mamta Singh, Pramod Kumar Pandey

**Affiliations:** College of Fisheries, Central Agricultural University (I), Lembucherra, Tripura, India

**Keywords:** *O. bimaculatus*, mitochondrial genome, phylogenetics

## Abstract

The complete mitochondrial genome of *Ompok bimaculatus* was obtained, using illumina high-throughput NextSeq 500 with 2 × 150 bp sequencing of mitochondrial DNA. The genome of *O. bimaculatus* was 16,482 bp in length (GenBank Accession No. KY887474) comprised of 13 protein-coding genes, 22 tRNA genes, 2 rRNA genes and a control region i.e. D-loop. In present mitogenome, 9 SSR were identified and validated *in silico* and secondary structures of all the 22 tRNA were predicted. The arrangement of genes was found identical to other siluriformes fish mitogenomes available in NCBI database. Phylogenetic relationship with closely related species were established which provide useful insights into taxonomic status of the species.

*Ompok bimaculatus* (Siluriformes: Siluridae), a butter catfish is locally known as ‘pabda’ in north-east India. Owing to the taste (Tripura delicacy), importance during customary rituals of marriage and child birth ceremony in Bengali community of Tripura and Near Threatened IUCN status (Ng et al. 2010), the fish was declared as state fish of Tripura in the year 2007. This species is widely distributed in Pakistan, India, Sri Lanka, Bangladesh and Myanmar. Overexploitation of this species for food is a major threat and has resulted in marked population decline (Mishra et al. 2009; Ng et al. 2010). The anthropogenic threat, its economic importance and IUCN red list status, warrants urgent need of taxonomic and phylogentic studies of the species.

For the present study, sample of *O. bimaculatus* were obtained from grow out pond of College of Fisheries (CAU), Tripura India (23^ã^54.248′ N, 91^ã^18.465′ E) in the month of November 2016 and maintained at the Fish Museum (specimen voucher no. OB-WM-TR01) of College of Fisheries (Central Agricultural University), Tripura, India. Total mitochondrial DNA was isolated from liver tissue and sequenced, using illumine high- throughput NextSeq 500 with 2 × 150 bp paired end chemistry. A total of 9,287,508 read were obtained and a reference guided assembly was done, choosing closest species i.e. *Pterocryptis cochinchinensis* (Genbank Accession No. NC02107). The mapping of reads to the reference was performed, using BEW-Mem and consensus sequence was generated with the help of Samtools (Li et al. 2009). Gene prediction and annotation of the assembled genome was done with the help of MitoAnnotator and SSR were identified using MISA ver. 1.0. Maximum likelihood (ML) Phylogenetic tree was constructed in MEGA ver. 7 (Kumar et al. 2016).

The complete mitochondrial genome of *O. bimaculatus* is 16,648 bp in length (GenBank Accession No. KY887474), comprising of 13 protein-coding genes, 22 tRNA genes, 2 rRNA genes and a 864 bp long control region i.e. D-loop. Majority of the genes were found on H strand, except ND6, tRNA^Glu^, tRNA^Pr^°, tRNA^Gln^, tRNA^Ala^, tRNA^Asn^, tRNA^Cys^, tRNA^Tyr^ and tRNA^Ser^ which were encoded on L strand. GC % of protein coding, tRNA, rRNA genes and D**-**loop region was found to be 43.03, 43.44, 45.34 and 38.58, respectively. A total of nine short sequence repeats (SSR) were identified and validated *in- silico*.

Phylogenetic relationship of *O. bimaculatus* was established with 20 closely related catfishes, using ML method based on the Kimura 2-parameter model (Kimura 1980). The tree with the highest log likelihood (−145997.4539) is shown as Figure 1. Initial tree(s) for the heuristic search were obtained automatically by applying Neighbor-Join and BioNJ algorithms to a matrix of pair-wise distances estimated, using the Maximum Composite Likelihood (MCL) approach, and then selecting the topology with superior log likelihood value. *Ompok bimaculatus* showed closest relationship with *Peterocryptis cochinchinensis* and clustered with other catfishes of family siluridae with the highest bootstrap support value, i.e. 100%. Present finding was observed to be concordant with a recent report of time calibrated mitogenome phylogeny of 20 catfish families (Kappas et al. 2016). Established phylogenetic relationship with closely related species will provide useful insights into taxonomic status of the economically important butter catfish *O. bimaculatus* and help in management and conservation of the species.

**Figure 1.**
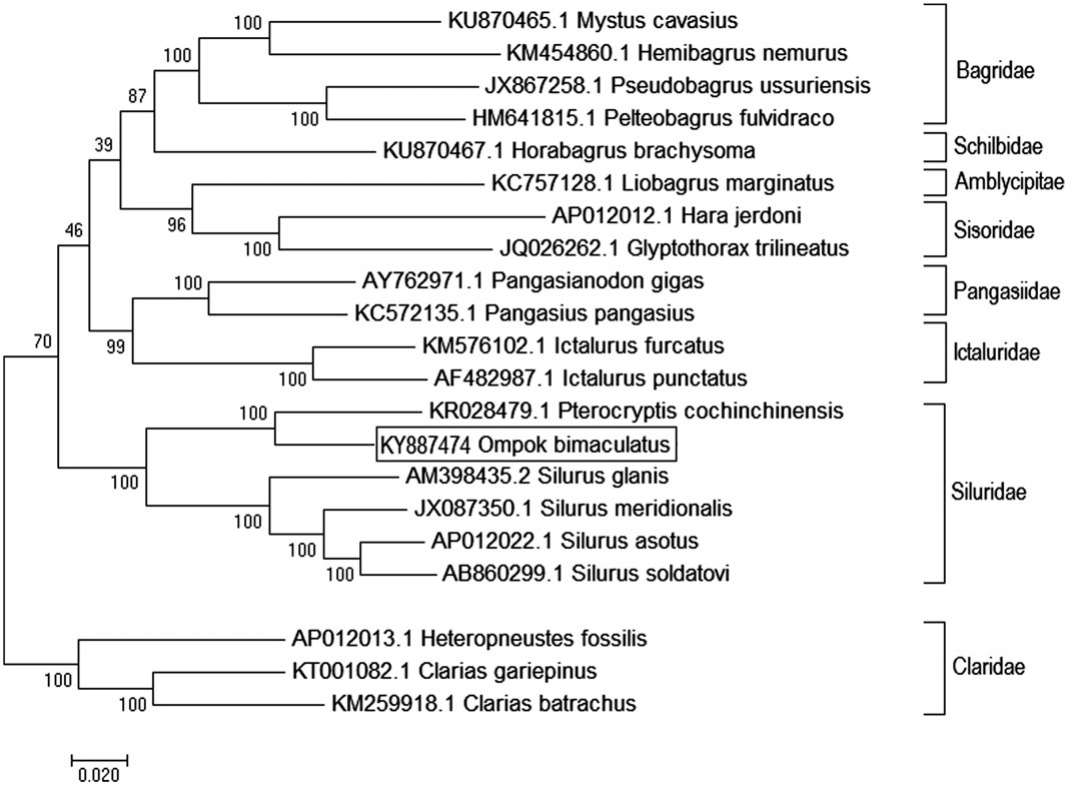
Maximum likelihood phylogenetic tree of closely related 21 catfishes. The percentage of trees in which the associated taxa clustered together is shown next to the branches and tree is drawn to scale, with branch lengths measured in the number of substitutions per site.
